# A perspective on bridging scales and design of models using low-dimensional manifolds and data-driven model inference

**DOI:** 10.1098/rsta.2016.0144

**Published:** 2016-11-13

**Authors:** Jesper Tegnér, Hector Zenil, Narsis A. Kiani, Gordon Ball, David Gomez-Cabrero

**Affiliations:** 1 Department of Medicine, Unit of Computational Medicine, Center for Molecular Medicine, Karolinska Institutet, Solna, Sweden; 2 Center for Molecular Medicine, Karolinska Institutet, L8:05, 171 76 Stockholm, Sweden; 3 Department of Medicine, Unit of Clinical Epidemiology, Karolinska University Hospital L8, 17176 Stockholm, Sweden; 4 Science for Life Laboratory, Stockholm, Sweden; 5 Mucosal and Salivary Biology Division, King's College London Dental Institute, London SE1 9RT, UK

**Keywords:** computational biology, modelling, living systems, big data, model reduction, systems biology

## Abstract

Systems in nature capable of collective behaviour are nonlinear, operating across several scales. Yet our ability to account for their collective dynamics differs in physics, chemistry and biology. Here, we briefly review the similarities and differences between mathematical modelling of adaptive living systems versus physico-chemical systems. We find that physics-based chemistry modelling and computational neuroscience have a shared interest in developing techniques for model reductions aiming at the identification of a reduced subsystem or slow manifold, capturing the effective dynamics. By contrast, as relations and kinetics between biological molecules are less characterized, current quantitative analysis under the umbrella of bioinformatics focuses on signal extraction, correlation, regression and machine-learning analysis. We argue that model reduction analysis and the ensuing identification of manifolds bridges physics and biology. Furthermore, modelling living systems presents deep challenges as how to reconcile rich molecular data with inherent modelling uncertainties (formalism, variables selection and model parameters). We anticipate a new generative data-driven modelling paradigm constrained by identified governing principles extracted from low-dimensional manifold analysis. The rise of a new generation of models will ultimately connect biology to quantitative mechanistic descriptions, thereby setting the stage for investigating the character of the model language and principles driving living systems.

This article is part of the themed issue ‘Multiscale modelling at the physics–chemistry–biology interface’.

## 1. Introduction

A fundamental challenge for most, if not all, scientists is how to advance from observations of data to a causal understanding of what goes on behind the scenes thereby coming to grips with the generative processes producing observations. Succeeding entails (some) predictive power because once the causes are known, their consequences and origins can be systematically investigated from the underlying physical laws, which in turn can be used for experimental tinkering, thereby assessing whether the consequences are in accordance or not with the predictions. Clearly, because correlations not necessarily imply causation, a scientific understanding of the world in terms of causes and effects is challenging as already stressed by major thinkers from Aristotle to Hume. With the scientific revolution gaining momentum in the seventeenth century, the notion of mathematical modelling using equations became the efficient language of choice to understand and predict events in the natural world [1]. Four hundred years later, we are in a situation where vast amounts of data, of a large variety, often referred to as Big Data are being produced from new technical platforms monitoring events in the natural world. Hence, there is urgency in developing and using advanced mathematics in order to process and in particular to understand the generative processes behind the data. Thus, we need theory in the form of equations capturing the causal generative processes [2–6]. In contrast with the established modelling culture in physics and in part in chemistry, biology is still to a great extent collecting and organizing empirical observations. However, with the accelerated data production capabilities in biosciences, we would argue that there is an emergent need to understand the biological systems producing the data beyond statistical correlations. Instead, of mathematical modelling we have witnessed the emergence of fields such as bioinformatics, focused on management, signal extraction and statistical and machine-learning based analysis of the large and diverse data [7]. The medical and biological communities are in this narrow sense forced to accept the infusion of computational techniques for data analysis. In practice, statistics and machine-learning methods have therefore become increasingly important to find patterns or correlations in complex datasets, whereas mechanistic mathematical modelling has not become mainstream.

In our view, we find two contrasting perspectives on the topic of mathematics in general and computational modelling in particular in biosciences. On the one hand, modelling living systems and diseases appears unrealistic; possibly, if we wait a century or two then we may have assembled all the facts and observations [8]. This is a viewpoint not uncommon in the life-science community in general and among medical doctors in particular. The idea being that we need to have ‘all’ the experimental facts on the table before even embarking upon such an ambitious modelling task. In short, this line of thinking could be one of the reasons why mathematical modelling is not and has not (yet) become mainstream within medicine and biology.

At the other end of the spectrum, we find the view that modelling biological systems is relatively speaking straightforward. As the governing equations of atomistic systems are well defined, there is no doubt that modelling is useful and interesting. This view comes through most clearly among investigators with training in the hard sciences such as physics, chemistry and mathematics.

In this short perspective review, we argue that techniques for analysing and reducing complex mathematical models, as developed in physics and chemistry, are useful for current modelling efforts in biology. To sum up, the existence of regularities in biology implies existence of manifolds in a geometrical sense. Furthermore, as modelling biological systems is challenging due to inherent modelling uncertainties such as deciding on the nature of the equations, which variables to select and how to determine model parameters, low-dimensional systems analysis will become an important tool in mitigating the uncertainties in modelling living systems. Finally, fuelled by rich molecular data in biology, we anticipate a new generative data-driven modelling paradigm in biology integrated with a low-dimensional manifold analysis of the governing equations. We conclude the paper by discussing the implications for our understanding of living systems.

## 2. Existence of effective low-dimensional manifolds: success of modelling of chemical systems from first fundamental quantum mechanical principles

From the standpoint of chemistry and physics, an atomistic perspective on living systems may appear not only desirable but also conceptually straightforward. The governing equations are known and the challenges rather include how to (numerically) perform multiscale simulations and how to interpret the extensive simulation results. Hence, the lack of diffusion of mechanistic mathematical modelling in biology and medicine may therefore appear perplexing from this point of view. However, here we would like to argue that the deep challenges, which have been addressed when modelling chemical systems from a fundamental physics basis, are not unlike the challenges ahead of us when performing ‘forward’ modelling of living systems. Here, we therefore ask what strategies could we learn from chemical systems modelling when modelling significantly larger collections of entities making up living systems? To this end, we will briefly review recent developments in computational chemistry.

From a quantum point of view, wave functions describing electrons and atomic nuclei are of primary interest, whereas a chemical viewpoint targets atoms or groups of atoms. A classical Newtonian modelling approach of chemical systems has fewer degrees of freedom compared with the complete underlying quantum mechanical description. This is not only a technical question of simulation speed but the important issue is how to ‘approximate’ a quantum mechanical description using a classical approach. In part, this is a technical numerical question but such an analysis necessarily involves considerations such as what parts and interactions at one scale make a difference at another scale, thus addressing what is the relevant coarse-graining given the system of investigation. Historically, some of the key ideas have been to use a detailed (quantum) description where necessary and to use approximation of the other parts. Important concepts in this development were the derivation of an effective potential covering both weak and strong interactions, in combination with guiding principles such as searching for energy minimization in order to reveal actual chemical configurations and structures. Larger chemical systems could then be viewed as being embedded in a dielectric continuum where computer intensive methods could be used to compute effective empirical potentials and energy minima, thus capturing the structure and dynamics of the system without requiring full-scale quantum mechanical simulations of the system. Yet, classical potential-based methods are as a rule only valid when interacting molecules are weakly perturbed or the number of elements considerably reduced. For example, the case when reactants produce new molecules is challenging to address with a classical approximation as this situation represents a strong perturbation. One concept is therefore to use a hybrid approach in the sense of performing quantum chemical modelling where necessary and then invoke those computations in a larger context handled by a classical formulation. These developments have led to several Nobel Prizes, where the latest in 2013 was on developments of multiscale methods for complex systems [9]. Conceptually, this is not too dissimilar to the idea in finite-element computations where increased numerical accuracy using a finer grid at those space coordinates where ‘interesting’ actions, or stress, takes place. See also related work on density functional theory and simulation [10].

From this brief overview, we emphasize two points. First, these advances in computational chemistry are not only technical but actually teach us principles of collective structure and dynamics across scales that actually are at work in nature. Hence, principles such as the existence of effective potentials, energy minimization, as discussed above, are fundamentally a reflection of the existence of natural laws, which make a shorter description of a system than itself possible. We have no reason to believe that this would not apply when modelling living systems. Hence, we should expect the existence of simpler models than the living systems themselves. We therefore conclude that we anticipate the existence of analogous effective potentials yielding sub-manifolds representing the surface upon which the effective dynamics in living systems takes place. We do acknowledge that logically it could well be the case that living systems are computationally irreducible in some sense, which inanimate systems are not. However, it is a reasonable working hypothesis that the modelling situation does not differ in such a fundamental manner at the border between living and non-living systems. For a different viewpoint, see for example the seminal work of Robert Rosen [11]. Second, while the existence of low-dimensional manifolds in nature is reassuring, it does not follow how to actually find such structures. In the case of physics, where we have the equations, and a chemical readout we can work out the computational connections between the levels if we have good insight into the specific problem. Yet, how to automatically in a machine driven manner discover the relevant features, i.e. to perform a coarse-graining of a system, is unclear. Another way to pose this question is to ask, which are the relevant order parameters for the problem at hand? In conclusion, the challenges and conceptual results when bridging scales between physics and chemistry models are expected not to be dissimilar when bridging scales between models of biological living systems and models of their underlying chemical parts and interactions.

## 3. Phenomenological modelling of biological systems at some scale in the face of uncertainties on equations, features and parameters

Now, turning to biological systems, encompassing significantly larger groups of atoms, the modelling situation is quite different when considering dynamical questions beyond structural biology. In essence, there is no fundamental ground, analogous to a quantum mechanical description underlying atomistic modelling and simulation in chemistry nor is it in any way feasible or desirable to aim in itself for an atomistic description of larger scales when modelling living systems. Here, we distinguish between whether it would in principle be conceivable to perform an atomistic model-based description of a living system versus whether it would be a useful approach in terms of understanding and deciphering governing principles of living systems. Independent of this distinction, we posit that the existence of lower dimensional manifolds would facilitate our understanding of living systems. However, it is unclear how to find such reduced descriptions in practice. We will return to this fundamental challenge in the concluding section in this paper. In practice, when modelling biological systems we are faced with at least three levels of uncertainty [5], which, we would argue, are more severe challenges when compared with the case when modelling chemical systems.

First, it is therefore fundamentally unclear what the governing equations are or should be when analysing a given part or subsystem of a living system. Hence, there are no first principles guiding us to what kind of equations which could/should be used or alternatively which are useful despite not being mechanistically faithful. From a physics standpoint, we could argue that differential equations are the proper language to describe nature. These could take the shape of ordinary differential equations capturing dynamics of point elements developing over time such as neural signal propagation [12]. Alternatively, partial differential equations could be used to describe reaction–diffusion and pattern formation in biological systems [13]. Yet, adopting a computer science/logical perspective, Boolean models [14] may appear useful whereas moving towards a statistical/machine learning perspective readily yields regression or classifier models to appear as natural language. Clearly, this issue depends on data at hand and the question addressed if we think of models as tools for understanding phenomena in nature. However, in analogy with physics, we may view the models as capturing a fundamental level of how these systems work, thus leading to considerations on what is an appropriate language (i.e. model) capturing living systems. Our point here is that this problem is in general fundamentally unresolved and the task of modelling living systems makes this a pertinent problem. For example, one could even put forward an argument that living (and other) systems are at their root information processing entities, thus advocating a language (model), which does not necessarily refer to the underlying physical entities, such as information theory in some shape. Yet, approaching this problem by for example striving to identify relevant and predictive differential equation models may prove a useful path forward, aiming to discover the fundamental information processing principles if there are any. This is necessarily a trial and error prone approach but because we do not have access to the fundamental laws in biology we have to try capturing the observed regularities using not only differential equations but rather assess a suite of model languages. Following this paradigm, such models can subsequently be systematically investigated. The overall point is that when trying to capture data with the wrong kind of model makes the model very complex and may lead us astray. For example, describing the temporal evolution of Heaviside functions using a Fourier basis would require numerous coefficients in order to capture the trajectory with a certain degree of numerical accuracy. In the case of chemical systems, we ‘know’ the form of the underlying equations whereas this is not the case for living systems.

Second, assuming that we could home in towards an appropriate formalism, the modeller is faced with a severe feature selection problem, which translates into the question of the identity of the relevant state variables for the system under investigation [15,16]. On the one hand, all the entities that are measured from the system could define the set of state variables to be represented. Such a model could easily become very complex and possibly a certain subset of combinations of measured state variables could be a more faithful or useful representation of the governing dynamics driving the system. On the other hand, the challenge of latent variables, entities not measured, but affecting the governing dynamics, introduces severe modelling challenges. For example, data hungry Bayesian modelling techniques assume as a rule faithfulness, i.e. the absence of latent variables [17]. In contrast with the case with chemical systems modelling, we can determine whether the macroscopic approximation is sufficiently good using the quantum equations as a reference. When the macroscopic model is good enough, we have arrived at a description which is amenable for deep analysis.

Third, on top of these two major principal problems, every computational model of living system comes equipped with a huge number of parameters, which cannot as a rule be measured or have not yet been measured experimentally. This is referred to as the parameter uncertainty problem. Last, inspecting available mathematical models in biology and medicine [18] reveals that they are as a rule very sensitive to perturbations either in the parameter space or in the model structure.

This situation has led to significant scepticism about the prospect of modelling in life science. On the face of it, the biological or medical investigator appears to have a strong case against using mechanistic models. We would like to argue that the impact of these principal challenges taken together makes the modelling in biology different in nature when compared with the situation in physics and chemistry. One way to deal with this situation in biology is to shift focus to a narrow well-defined system where the investigator has knowledge about the relevant state variables, and can perform targeted experiments to estimate parameters of the system. A paradigmatic historical success story, awarded with a Nobel Prize, is the elucidation of the chemical basis of the propagation of a nerve impulse, referred to as an action potential in neurons.

This work, carried out in the mid twentieth century, prior the molecular revolution in life science, has in practice defined how to do modelling in biology. The classical model of Hodgkin & Huxley [19] of the action potential in the Atlantic squid giant axon was indeed a milestone. The action potential is used for communicating signals (information) over large distances when the potential propagates over the axon, modelled by a set of coupled nonlinear differential equations associated with several membrane channels with specific time- and voltage-dependent properties. The Hodgkin–Huxley model of the membrane potential in the squid giant axon provides an informative example of both the useful and artificial aspects of model building. The squid giant axon is itself a model system; it is sufficiently large (0.5 mm) to allow access for electrodes while still being a close analogue of conventional-scale neurons. The potential across the axonal membrane is a consequence of relative concentrations of sodium and potassium ions, and time variation is caused by the relative rate of active transport into the cell versus ion loss through leakage and gated channels, plus a small contribution from the capacitance of the lipid bilayer itself. The complex spiking behaviour of neurons largely derives from the nonlinear behaviour of voltage-gated channels. Hodgkin & Huxley were able to identify separate voltage-dependent activation and inactivation effects in these channels, which explain refractory effects that earlier models added ‘by hand’. They also determined that the ion species had different dynamics and the overall potential needed to be the sum of distinct contributions. The resulting set of ordinary differential equations successfully describe both single activation and spike-train generation behaviours observed in nature.

This illustrates a paradox of modelling; the Hodgkin–Huxley model provides a very useful description of an axon. They successfully identified the minimal set of processes they needed to include to produce a faithful model, but their parametrizations tell us little about the actual underlying mechanisms. Conversely, detailed knowledge of the actual membrane channel proteins would not present an easy starting point to reproducing the macroscopic behaviour the model describes. This remarkable piece of work has defined a modelling paradigm in biology. It provided a coherent explanatory framework, accounting for several observations regarding the role(s) of ionic flows leading to a predicted speed of propagation of the action potential. Notably, this was achieved without a molecular description of the underlying circuitry in terms of ion channels and the internal organization of the cell. Moreover, it was not based on or depended upon a fundamental quantum mechanical description of the system. On the contrary, the approach was in essence classical in a physical sense, where the axon could be viewed as firm phenomenological ground to be investigated. This has not only defined a framework for the analysis and modelling of excitable cells across biology but also served as a paradigm in the analysis of cell differentiation, apoptosis and numerous other cellular dynamic processes in biology. Luckily, or rather because of a clever choice of model system, there were no other strong correlations at the microscopic level in the axon that interfered with the governing dynamics of the four Hodgkin–Huxley equations, which were sufficient to account for the experimental observations. However, the reason for success, the choice of a narrow model system, also entails the reason for the limited generality and usefulness when investigating larger systems. Clearly, to investigate large (neural) systems, the number of equations and parameters explode. We would argue that the few success stories of modelling in biology share these characteristics. To overcome the three faces of uncertainty, discussed above, the strategy has been to investigate a small system, in close conjunction with an experimental set-up, where there are good arguments defining a phenomenological basis which should be taken for granted, i.e. to avoid the myriad of molecular, chemical complexities down to the level of quantum mechanics. The main problem with these success stories is that they do not readily scale to either other or larger systems. In parallel, during the last decade there has been ambitious projects targeting whole-body models, or a virtual physiological human, as a paradigm [20]. We will return to this issue in the concluding section reflecting upon whether small-scale bottom-up modelling is the only way forward when quantifying living systems.

## 4. From forward modelling and finding manifolds to inverse techniques for model design: a pending synthesis?

Now, examining biological systems in greater detail or investigating larger systems produces complex nonlinear phenomenological models. All such large models are as a rule afflicted by the three issues of model formalism, variable selection and parameter problem, yielding fragile and sensitive models. We would like to argue that this state of affairs in combination with the scarcity of validated predictions from modelling work has created scepticism towards modelling in biology in general and medicine in particular. Yet, there has been and is currently a flurry of work within the computational communities on developing tools for understanding, analysing, and constructing these kinds of phenomenological biological models.

In brief, model reduction has been a recurring theme across several sub-fields of computational biology and neuroscience with special reference to dynamical forward models. The idea being that if a complex nonlinear model can be reduced in complexity (fewer state variables and parameters), then the investigator can more readily discern which parameters and state variables are more crucial for the model behaviour, thus facilitating model analysis and understanding. One example is the reduction of the four-dimensional Hodgkin–Huxley model to a two-dimensional FitzHugh–Nagumo (FHN) system [21]. The core idea is to perform a time-scale separation into a fast and slow subsystem [22]. This has been used in a number of model reduction studies including the cell cycle [23,24]. Such a reduction facilitates the identification of critical parameter(s) in the system, often related to how the fast system is being slaved by the slow state variable, corresponding to a slow manifold. This mode of analysis has also been extended to more complex chemical models and a large body of work is currently addressing how to identify the slow invariant manifold in more complex high-dimensional nonlinear systems [25]. In the case of neuron models, extensive analytical work has resulted in effective low-dimensional models such as leaky integrate-and-fire (LIF) neuron model [26]. These theoretical advancements have made it feasible to construct larger circuit models using Hodgkin--Huxley, FHN or LIF models of the individual neurons, depending on the needed level of detail required for the specific systems analysis at hand. It should be stressed that such phenomenological circuit models have provided important insights despite that they are not grounded in faithful molecular representations. However, to gain such insights, beyond simply numerically solving these models repeatedly for different parameter values, theory has been essential [27]. Again, theory in this context means techniques for model reduction in order to identify the critical elements in the circuit model. The second major idea is to employ and to develop modified versions of mean field techniques for computing effective smaller models representing and capturing the ‘essential’ system dynamics occurring in the full-scale yet simplified model from a biological standpoint [28]. For example in [29], the authors derived explicit analytical expressions in a neural working memory circuit model, for the capacity to hold memories over time, where the original system was defined by thousands of nonlinear Hodgkin--Huxley equations.

In essence, these two complementary approaches of slow manifold computation and mean field reduction have been instrumental to facilitate analytical insights as well as enabling large-scale models without being forced to rely entirely on brute force numerical calculations. Yet, it is difficult to generically apply these techniques because in many cases specific adaptations have to be made depending on the equations at hand and depending on what the scientific question is. Conceptually, these methods effectively accomplish a coarse-graining of a complex nonlinear system. It is an open question whether such coarse-graining should be viewed as a practical tool to understand a complex system, or if a natural living system actually makes use of the effective reduced system dynamics in order to be more efficient in operating as a fast, adaptive living system capable of predicting future events and actions. Here, we like to highlight the conceptual similarity with the theoretical and numerical work referred to in our first section on bridging between a quantum mechanical description of a chemical system and effective equations incorporating dynamics and structure of groups of atoms. Yet, forward modelling and analytic dissection have been less visible in the computational biology community when compared with investigators working in computational neuroscience. Part of the reason being that experimental techniques in neuroscience such as recording electrical and chemical activity over time lend themselves naturally to ask dynamical questions, thus requiring time-dependent models to understand the generative processes. This is possibly even clearer in the chemistry community. By contrast, the molecular revolution in life science fuelled by rapid advances in technical capabilities in sequencing and molecular profiling of living systems, from tissues to single cells, has as a rule generated more static data. This has created a situation where inverse problems have had a higher priority on the research agenda in computational biology, in preference to forward explicit dynamical modelling. The generic situation could be described as given a set of input data, infer a (statistical) model consistent with the observations. Examples include (i) from a string of amino acids to inference of the folding structure (http://predictioncenter.org/), (ii) from a set of DNA sequences reconstruct (infer) the evolutionary tree consistent with the observations [30], and (iii) from a set of molecular profiles (expressed genes, proteins, metabolites) to reconstruct (infer) a graph representing a molecular interaction network [31,32]. As our understanding of the governing equations in each of these examples from first principles is limited, statistical and machine learning techniques have become very important in addressing the corresponding inverse inference challenges in different domains.

In summary, there is a gap within the bioinformatics and computational biology communities between forward modelling approaches assuming a phenomenological ground including a form of the governing equations versus inverse problems, which in contrast are heavily dominated by statistical and machine-learning related techniques. Here, we find it evident that it would be valuable if this gap could be mitigated. Merging rich molecular data with powerful forward modelling techniques holds the promise to uncover deep principles of relevance for addressing Schrödinger's quest on ‘what is life’.

To this end, we think that two emerging trends will become important in bridging this gap. On the one hand, advances in molecular profiling techniques and experimental designs in the analysis of either populations or single cells are increasingly producing temporal data reflecting transitions between different biological states or effects of external perturbations. Examples include work in stem cell biology on how to either reprogram stem cells into specialized niches or transform a differentiated patient-derived cell like a fibroblast into therapeutically relevant cells such as dopamine-producing cells. Effects of drugs will increasingly also be analysed in a temporal mechanistic context. Thus, we anticipate that forward modelling techniques will enter these and other areas in biology. This is similar to the development over the last two decades in neuroscience where increasing amounts of complex temporal data have fuelled theoretical and computational neuroscience. Secondly, the molecular revolution in life science produces what could slightly misleadingly be classified as big data. Large datasets without a fundamental grasp of the generative models are as a rule interrogated with machine learning techniques in combination with statistical tools. Examples of this growing paradigm are abundant in leading biomedical journals. Yet, in our hands we would rather characterize these datasets as heterogeneous, defined by their multitude of different data types, each relatively small, in contrast with referring to them as ‘big data’. This view entails that the notion of data-integration becomes crucial in order to connect observations at different scales [33,34]. The data typically include genetic (DNA) information, activities of expressed genes, proteins and metabolites, in cells, tissues, organs and clinical data in human or experimental model systems. Clearly, data-driven methods have been and are currently being developed to fuse and integrate such diverse data. However, we anticipate that model-based methods will become crucial in order to link and comprehend rich and different molecular data types sampled from a given system. In summary, these two developments of increased production to complex temporal data, involving several data types, need model-based tools for analysis, thus in the end mitigating the gap between inverse and forward modelling approaches in biology. Given such a development, we expect model reduction techniques as previously discussed to become central in biology in general and computational biology in particular.

## 5. Molecular and atomistic simulations of small living systems

Thus far, we have reviewed and discussed modelling work from quantum mechanics to more complex chemical systems, and the current status of both forward modelling and inverse data analysis in biology. We highlighted the similarities in the necessity of identifying governing principles on how to bridge across scales as well as delineating their domains of validity. Yet, one flagrant gap is between efforts of modelling complex chemical systems, with a rigorous foundation, and approaches in biology, which assumes a phenomenological basis for constructing more complex and larger models. We can therefore ask to what extent can we account for a biological system in terms of collective dynamics of atoms subserving nonlinear chemical dynamics?

Interestingly, there have been a couple of proof-of-principle studies demonstrating that by collecting and integrating available molecular data into computational models, accurate predictions of interventions into the system can actually be produced. For example, a computational model [35] of *Halobacterium salinarum* NRC-1 was first constructed through massive data integration and machine learning driven inference of the regulatory network, a graph representing the molecular components and their interactions in the system. Next, using changes in more than 70 transcription factors in relation to a dozen environmental factors, the investigators could on the basis of the model, predict dynamic transcriptional responses of all these genes in close to new experiments. The authors concluded that their approach could be used to construct similar kinds of models for any organism using only a modest number of experiments. As second example of similar nature is the ambitious whole-cell computational model of the life cycle of the human pathogen *Mycoplasma genitalium* [36]. The model accounted for all annotated gene functions and was validated against a broad range of data. Now, the model encompasses approximately 500 genes and their interactions. To deal with the different data types (DNA, RNA, proteins, metabolites, environmental conditions), the authors had to resort to a ‘manual’ coarse-graining in the sense of sub-dividing the system into 28 modules on the basis of our current understanding. Overall, these two examples demonstrate the feasibility of formulating predictive yet mechanistically faithful models of complete but small living systems. However, these efforts are by no means automatic and still require substantial domain knowledge on how to integrate different data types, and how to coarse grain the system. Despite this significant advance, we are faced with model and parameter uncertainties, and how to effectively understand or identify the governing manifold(s) of these highly complex nonlinear models. Researchers in Japan have taken this second example one step further in that they have produced a complete atomistic model of the cytoplasm in *Mycoplasma genitalium*, thereby integrating an atomistic physics description with biochemistry [37]. They used a known metabolic reaction network as a basis, then replacing the components with their respective atomistic representation. While the model bridges by its very construction several spatial scales, it is not clear how to validate the simulation results. However, their impressive work demonstrates a possible road to grounding phenomenological biological models towards an atomistic level thereby making contact with a representation of groups of atoms, in principle derivable from a quantum point of view. There are other emerging examples of this style of modelling, such as the prediction of nucleosome positioning using atomistic energy minimization principles [38].

This bottom-up style of modelling raises a couple of issues for discussion. First, it should be noted that the models themselves do not necessarily provide insights, in part due to their complexity. To wit, why and how are these models working, which aspects are essential and which are not? The advantage is that the models can be systematically investigated using model reduction techniques in order to grasp the effective potentials or sub-manifolds driving these systems. The outcome of such an analysis may be the design of smaller effective models capturing the dynamics of these systems, thereby possibly discovering design principles. Complementary, in such smaller systems we can also ask whether other model languages including information-based approaches could equally well capture the function of these systems. For example, using appropriate reduction techniques applied to different models capturing phenomenological regularities expressed in different formalisms may converge to a common core of dynamical design principles. These could in turn possibly be expressed in different terms using information theory. The outcome of such a programme could be a view of living systems, which is less dependent on the material basis, akin to the standpoint of functionalism in philosophy of mind. Last, we may also define or discover quantities operating at the level of organisms, which are not visible at the microscopic scale. This does not exclude that such quantities are in principle derivable from ensemble of microscopic variables, in analogy with the concept of temperature in statistical mechanics

## 6. Concluding remarks: the modelling landscape and beyond

**Figure 1.**
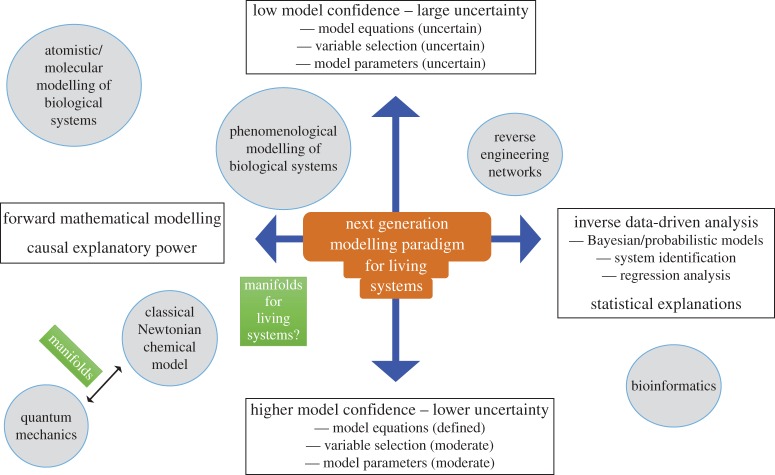
A schematic illustration of the modelling landscape in physics, chemistry and biology. The horizontal axis represents the spectrum from forward explicit modelling of a system to the purely data-driven statistical analysis. Our degree of certainty of the models and the outcomes is captured by the vertical axis.

Figure 1 summarizes schematically the overall modelling landscape as presented in this brief review. Here, we organize the different activities along two axes. On the one hand (left-hand side of the horizontal axis), mechanistic models can have a strong explanatory power in revealing causes and consequences of different interventions, thus being predictive with respect to mechanisms. At this end, we find bottom-up modelling approaches such as fundamental physics and atomistic modelling of chemistry and biology. At the other end at the horizontal axis, we have purely data-driven analysis with an emphasis on pattern and correlation detection. Such models may be predictive in the sense that if we observe some variables we can readily expect other combinations of variables to co-occur due to correlations or statistical relationships. This is useful in a biomedical biomarker context. The bulk of bioinformatics research and development dealing with molecular data in biology is essentially of this kind. In between these two extremes, we first find what is referred to as reverse engineering or inference of data into a static network model or a graphical probabilistic model [39]. This approach may suggest causal relationships between the variables. This is a major activity occurring in research areas also referred to as network biology or systems biology. Phenomenological modelling on the other hand makes explicit representation of mechanisms, in a small or large model, and as discussed previously being more or less valid depending on the assumptions, but eventually aiming for causal explanations and predictions of the effects of an intervention. The other vertical dimension represents the confidence or the certainty we have in what comes out from these different approaches. Note that when we state that we have low confidence in a model and the output, i.e. large uncertainty (top of vertical axis), it may depend on different combinations and aspects of the uncertainty. For example, we can be very confident about the nature of equations, but less certain how to determine the model parameters. In large-scale atomistic simulations of biological systems, we can readily represent the dynamical equations at the atom scale but it is less clear how to determine the parameters of the active biological system when there is a surrounding environment such as water and being situated in a cellular context. At the other end (right-hand side of the horizontal axis), using a statistical model while we could compute confidence intervals for parameters given the data, the subset of predictive variables are easily underdetermined and there may be latent variables outside the model. In summary, there are several sources of such fundamental uncertainty in addition to technical variability of the data itself. As illustrated in the figure, both phenomenological modelling and reverse engineering techniques are afflicted with strong uncertainties. For example, comparing close to 40 different reverse engineering algorithms on a reference dataset revealed that each of them captured different yet incomplete aspects of the regulatory gene network [40]. Furthermore, it is important to clarify which algorithms preserve or capture which aspects of the generative network [41,42]. Thus, our analysis clearly highlights the need for development of modelling techniques with increased causal explanatory power where we must reduce the uncertainties dramatically. Now, at this point it would be tempting to believe that more data will solve this challenge. More data are good but measuring more entities also increases the search space of putative causal interactions, thus easily leading to larger models where we would still be in an unfavourable low data-to-model-complexity ratio. We would therefore like to argue that we need other complementary ideas such as top-down driven constraints, in order to tame the combinatorial complexity when considering huge sets of molecules. It is a completely open issue whether this is a good approach and how to find such principles. Interestingly, there appears to be an emerging literature in this direction using ideas from compressed sensing and sparse constraints (e.g. [43–45]). There is a very informed discussion of these issues in [46,47]. Here, we have argued that using technical tools and insights derived from modelling reduction analysis, as practiced in computational chemistry and neuroscience, we may find hints on how to search for effective models. In our hands, what is needed is an integration of a data-driven approach identifying the effective components spanning the low-dimensional space in the data, in combination with the functional mathematical form of the expected manifold. We refer to such a development as the next generation modelling paradigm for living systems (centred at the origin of figure 1). In summary, we think this representation of the modelling landscape of selected parts across physics, chemistry and biology indicates where we need to increase efforts and progress in order to bridge between what can be perceived as modelling silos.

Next, we would like to clarify that our view on modelling is agnostic with respect to the issue of reductionism. The vantage point is that because nature is connected, events at different spatial and temporal scales are related to each other, within some limits naturally. Networks in biology are one example of this principle [48]. Hence, models capturing nearby levels (i.e. quantum descriptions of chemical systems) will thereby be amenable for model reduction and mapping between state variables. This view is also consistent with the emergence of new phenomena at one level not perceivable at another level, such as the example of the concept of temperature. This elementary observation together with the existence of physical laws is the basis for the fact that a system in nature allows a shorter description than the system itself. Further supporting this perspective is the crucial derivation by Noether [49] that there exists a conservation of an entity whenever there is a symmetry in the system. This has been exploited and extended in physics, essentially leading to a global constraint on which states a system can visit, hence an effective reduction in complexity. Whether there are analogous yet modified notions of symmetry in living systems remains to be investigated. In summary, from the existence of low-dimensional manifolds, we conjecture that there exists a reduced system description in terms of complexity. Finally, when auxiliary bridges and modelling techniques can be developed as outlined above with applications across biology, chemistry and physics, we will be in a position to analyse these effective models of living systems from different vantage points. On the one hand, we can try to extract simplifying principles for different systems at different scales and to assess their respective validity. At this juncture, there are several promising candidate principles, which are discussed and investigated in different systems and contexts. These include effective potentials, free energy minimization, maximum entropy, computational mechanics, maximum calibre, motifs in biological networks, self-organization, compressed sensing, evolution as thermodynamics, algorithmic complexity and algorithmic laws of information [47,50–64]. These could be considered as deep principles or algorithms that systems in nature use or from another perspective that represent efficient machine-learning principles, which may the reason why some of them are actually used in nature.

It is out of the scope to review all these concepts and their applications. Here, we remark on the difference between *a priori* assuming a principle and then using it for constraining a given modelling task versus performing detailed modelling at different levels, and thereby discovering which principles are at work in which contexts in nature. We have in this review stressed the discovery aspects, i.e. revealing effective manifolds, which can readily be used as tools to validate and test various proposed principles such as those listed above.

## References

[1] Wigner EP 1960 The unreasonable effectiveness of mat hematics in the natural sciences. Commun. Pure Appl. Math. 13, 1–14. (10.1002/cpa.3160130102)

[2] Ara PM, James RG, Crutchfield JP 2016 The elusive present: hidden past and future dependency and why we build models. Phys. Rev. E 93, 022143 (10.1103/PhysRevE.93.022143) 26986324

[3] Wolkenhauer O, Green S 2013 The search for organizing principles as a cure against reductionism in systems medicine. FEBS J. 280, 5938–5948. (10.1111/febs.12311) 23621685

[4] Wolkenhauer O 2014 Why model? Front. Physiol. 5, 21 (10.3389/fphys.2014.00021) 24478728 PMC3904180

[5] Geris L, Gomez-Cabrero D (eds). 2016 Uncertainty in biology. Berlin, Germany: Springer (doi:10.1007/978-3-319-21296-8)

[6] Pearl J 2015 Causal thinking in the twilight zone. Technical report, no. R-456. Los Angeles, CA: UCLA Cognitive Systems Laboratory.

[7] Tegner J, Abugessaisa I 2013 Pediatric systems medicine: evaluating needs and opportunities using congenital heart block as a case study. Pediatr. Res. 73, 508–513. (10.1038/pr.2013.19) 23370412

[8] Tegnér JN et al. 2009 Computational disease modeling—fact or fiction? BMC Syst. Biol. 3, 56 (10.1186/1752-0509-3-56) 19497118 PMC2697138

[9] Karplus M 2014 Development of multiscale models for complex chemical systems: from H+H_2_ to biomolecules (Nobel lecture). Angew. Chem. Int. Ed. 53, 9992–10 005. (10.1002/anie.201403924) 25066036

[10] Van MT, Bühl M, Gaigeot M-P 2014 Density functional theory across chemistry, physics and biology subject areas. Phil. Trans. R. Soc. A 372, 20120488 (10.1098/rsta.2012.0488) 24516181 PMC3928866

[11] Rosen R 1993 On models and modeling. Appl. Math. Comput. 372, 359–372. (10.1016/0096-3003(93)90128-2)

[12] Markram H et al. 2015 Reconstruction and simulation of neocortical microcircuitry. Cell 163, 456–492. (10.1016/j.cell.2015.09.029) 26451489

[13] Turing AM 1990 The chemical basis of morphogenesis. Bull. Math. Biol. 52, 153–197. (10.1007/BF02459572) 2185858

[14] Wang R-S, Saadatpour A, Albert R 2012 Boolean modeling in systems biology: an overview of methodology and applications. Phys. Biol. 9, 055001 (10.1088/1478-3975/9/5/055001) 23011283

[15] Nilsson R, Björkegren J, Pena J, Tegnér J 2007 Consistent feature selection for pattern recognition in polynomial time. J. Mach. Learn. Res. 8, 589–612.

[16] Fu S, Desmarais MC 2010 Markov blanket based feature selection: a review of past decade. Proc. World Congr. Eng. 1, 321–328.

[17] Lagani V, Triantafillou S, Ball G, Tegnér J, Tsamardinos I 2016 Probabilistic computational causal discovery for systems biology. In Uncertainty in biology (eds L Geris, D Gomez-Cabrero), pp. 33–77. Berlin, Germany: Springer (10.1007/978-3-319-21296-8_3)

[18] Chelliah V et al. 2015 BioModels: ten-year anniversary. Nucleic Acids Res. 43, D542–D548. (10.1093/nar/gku1181) 25414348 PMC4383975

[19] Hodgkin AL, Huxley AF 1990 A quantitative description of membrane current and its application to conduction and excitation in nerve. Bull. Math. Biol. 52, 25–71. (10.1007/BF02459568) 2185861

[20] Hunter P et al. 2013 A vision and strategy for the virtual physiological human: 2012 update. Interface Focus 3, 20130004 (10.1098/rsfs.2013.0004) 24427536 PMC3638492

[21] FitzHugh R 1961 Impulses and physiological states in theoretical models of nerve membrane. Biophys. J. 1, 445–466. (10.1016/S0006-3495(61)86902-6) 19431309 PMC1366333

[22] Rinzel J 1985 Excitation dynamics: insights from simplified membrane models. Federation Proc. 44, 2944–2946. 2415401

[23] Eriksson O, Andersson T, Zhou Y, Tegnér J 2011 Decoding complex biological networks—tracing essential and modulatory parameters in complex and simplified models of the cell cycle. BMC Syst. Biol. 5, 123 (10.1186/1752-0509-5-123) 21819620 PMC3176200

[24] Novák B, Tyson JJ 2008 Design principles of biochemical oscillators. Nat. Rev. Mol. Cell Biol. 9, 981–991. (10.1038/nrm2530) 18971947 PMC2796343

[25] Showalter K, Epstein IR 2015 From chemical systems to systems chemistry: patterns in space and time. Chaos 25, 097613 (10.1063/1.4918601) 26428566

[26] Gerstner W, Kistler WM, Naud R, Paninski L 2014 Neuronal dynamics: from single neurons to networks and models of cognition. Cambridge, UK: Cambridge University Press.

[27] Abbott LF 2008 Theoretical neuroscience rising. Neuron 60, 489–495. (10.1016/j.neuron.2008.10.019) 18995824

[28] Roudi Y, Hertz J 2011 Mean field theory for nonequilibrium network reconstruction. Phys. Rev. Lett. 106, 048702 (10.1103/PhysRevLett.106.048702) 21405370

[29] Edin F, Klingberg T, Johansson P, McNab F, Tegnér J, Compte A 2009 Mechanism for top-down control of working memory capacity. Proc. Natl Acad. Sci. USA 106, 6802–6807. (10.1073/pnas.0901894106) 19339493 PMC2672558

[30] Yang Z, Rannala B 2012 Molecular phylogenetics: principles and practice. Nat. Rev. Genet. 13, 303–314. (10.1038/nrg3186) 22456349

[31] Tegnér J, Björkegren J 2007 Perturbations to uncover gene networks. Trends Genet. 23, 34–41. (10.1016/j.tig.2006.11.003) 17098324

[32] Hill SM et al. 2016 Inferring causal molecular networks: empirical assessment through a community-based effort. Nat. Methods 13, 310–318. (10.1038/nmeth.3773) 26901648 PMC4854847

[33] Gomez-Cabrero D et al. 2014 Data integration in the era of omics: current and future challenges. BMC Syst. Biol. 8, 11 (10.1186/1752-0509-8-S2-I1) PMC4101704 25032990

[34] Tan K, Tegner J, Ravasi T 2008 Integrated approaches to uncovering transcription regulatory networks in mammalian cells. Genomics 91, 219–231. (10.1016/j.ygeno.2007.11.005) 18191937

[35] Bonneau R et al. 2007 A predictive model for transcriptional control of physiology in a free living cell. Cell 131, 1354–1365. (10.1016/j.cell.2007.10.053) 18160043

[36] Karr JR et al. 2012 A whole-cell computational model predicts phenotype from genotype. Cell 150, 389–401. (10.1016/j.cell.2012.05.044) 22817898 PMC3413483

[37] Feig M, Harada R, Mori T, Yu I, Takahashi K 2015 Complete atomistic model of a bacterial cytoplasm for integrating physics, biochemistry, and systems biology. J. Mol. Graph. Model. 58, 1–9. (10.1016/j.jmgm.2015.02.004) 25765281 PMC4388805

[38] Minary P, Levitt M 2014 Training-free atomistic prediction of nucleosome occupancy. Proc. Natl Acad. Sci. USA 111, 6293–6298. (10.1073/pnas.1404475111) 24733939 PMC4035975

[39] Villaverde AF, Banga JR 2013 Reverse engineering and identification in systems biology: strategies, perspectives and challenges. J. R. Soc. Interface 11, 20130505 (10.1098/rsif.2013.0505) 24307566 PMC3869153

[40] Marbach D et al. 2012 Wisdom of crowds for robust gene network inference. Nat. Methods 9, 796–804. (10.1038/nmeth.2016) 22796662 PMC3512113

[41] Zenil H, Kiani N, Tegnér J 2015 Quantifying loss of information in network-based dimensionality reduction techniques. J. Complex Netw. 4, 342–362. (10.1093/comnet/cnv025)

[42] Kiani NA, Zenil H, Olczak J, Tegnér J 2016 Evaluating network inference methods in terms of their ability to preserve the topology and complexity of genetic networks. Semin. Cell Dev. Biol. 51, 44–52. (10.1016/j.semcdb.2016.01.012) 26851626

[43] Brunton SL, Proctor JL, Kutz JN 2016 Discovering governing equations from data: sparse identification of nonlinear dynamical systems. Proc. Natl Acad. Sci. USA 113, 3932–3937. (10.1073/pnas.1517384113) 27035946 PMC4839439

[44] Schmidt M, Lipson H 2009 Distilling free-form natural laws from experimental data. Science 324, 81–85. (10.1126/science.1165893) 19342586

[45] Gomez-Cabrero D, Compte A, Tegner J 2011 Workflow for generating competing hypothesis from models with parameter uncertainty. Interface Focus 1, 438–449. (10.1098/rsfs.2011.0015) 22670212 PMC3262450

[46] Bialek W 2012 Biophysics: searching for principles. Princeton, NJ: Princeton University Press.

[47] Bialek W 2015 Perspectives at the interface of physics and biology. (http://arxiv.org/abs/1512.08954)

[48] Barabási A-L, Oltvai ZN 2004 Network biology: understanding the cell's functional organization. Nat. Rev. Genet. 5, 101–113. (10.1038/nrg1272) 14735121

[49] Buchwald JZ, Berggren JL, Fraser C, Sauer T, Shapiro A 2010 The Noether theorems. Berlin, Germany: Springer.

[50] Saunders M, Voth G 2013 Coarse-graining methods for computational biology. Annu. Rev. Biophys. 42, 73–93. (10.1146/annurev-biophys-083012-130348) 23451897

[51] Presse S, Ghosh K, Lee J, Dill KA 2013 Principles of maximum entropy and maximum caliber in statistical physics. Rev. Mod. Phys. 85, 1115–1141. (10.1103/RevModPhys.85.1115)

[52] Barnett N, Crutchfield JP 2015 Computational mechanics of input–output processes: structured transformations and the *ε*-transducer. J. Stat. Phys. 161, 404–451. (10.1007/s10955-015-1327-5)

[53] Zenil H 2012 Information theory and computational thermodynamics: lessons for biology from physics. Information 3, 739–750. (10.3390/info3040739)

[54] England JL 2013 Statistical physics of self-replication. J. Chem. Phys. 139, 121923 (10.1063/1.4818538) 24089735

[55] Gauvrit N, Zenil H, Tegner J 2015 The information-theoretic and algorithmic approach to human, animal and artificial cognition. (http://arxiv.org/abs/1501.04242)

[56] Alon U 2007 Network motifs: theory and experimental approaches. Nat. Rev. Genet. 8, 450–461. (10.1038/nrg2102) 17510665

[57] Tkačik G, Bialek W 2014 Information processing in living systems. PLoS Comput. Biol. 10, e1003408 (10.1371/journal.pcbi.1003408) 24391485 PMC3879139

[58] Candes E, Romberg J, Tao T 2005 Stable signal recovery from incomplete and inaccurate measurements. (https://arxiv.org/abs/math/0503066)

[59] Friston K 2010 The free-energy principle: a unified brain theory? Nat. Rev. Neurosci. 11, 127–138. (10.1038/nrn2787) 20068583

[60] Friston K 2012 A free energy principle for biological systems. Entropy 14, 2100–2121. (10.3390/e14112100) 23204829 PMC3510653

[61] Nussinov R, Wolynes PG 2014 A second molecular biology revolution? The energy landscapes of biomolecular function. Phys. Chem. Chem. Phys. 16, 6321–6322. (10.1039/c4cp90027h) 24608340

[62] Hall BK, Laubichler MD, Conrad H 2008 Waddington: towards a theoretical biology. Biol. Theory 3, 233–237. (10.1162/biot.2008.3.3.233)

[63] Zenil H, Kiani NA, Tegnér J 2016 Methods of information theory and algorithmic complexity for network biology. Semin. Cell Dev. Biol. 51, 32–43. (10.1016/j.semcdb.2016.01.011) 26802516

[64] Li Q et al. 2016 Dynamics inside the cancer cell attractor reveal cell heterogeneity, limits of stability, and escape. Proc. Natl Acad. Sci. USA 113, 2672–2677. (10.1073/pnas.1519210113) 26929366 PMC4790994

